# TB-related catastrophic costs and associated factors for patients in Ethiopia

**DOI:** 10.5588/ijtldopen.24.0230

**Published:** 2024-08-01

**Authors:** Z.G. Dememew, A.A. Deribew, D.G. Datiko, K. Melkieneh, T.G. Laloto, S. Negash, C. Gilmartin, M. Melese, P.G. Suarez

**Affiliations:** ^1^ USAID Eliminate TB Project, Management Sciences for Health, Addis Ababa, Ethiopia;; ^2^ Management Sciences for Health, Arlington, VA, USA

**Keywords:** tuberculosis, decentralized healthcare, DOT, directly observed treatment, out-of-pocket expenses

Dear Editor,

TB predominately affects the disadvantaged and the most vulnerable communities in precarious socioeconomic situations, with vicious cycles of poverty and disease.^1^ Despite the widespread adoption of ‘free TB care’ policies, TB-affected households still face prohibitive direct medical costs.^2^ TB illness is often compounded by high out-of-pocket (OOP) spending for direct costs (i.e., costs for diagnosis, treatment, transportation, and temporary accommodations when visiting health facilities) and indirect costs incurred due to the income foregone when looking for and receiving treatment or lost employment due to disability, stigma or discrimination.^1^ Catastrophic cost, defined as an OOP spending of more than 20% of a household’s annual income, forces many patients and their families to cut down on their daily necessities.^1,3,4^ TB services are exempted in Ethiopia, and yet the proportion of TB patients facing TB-related catastrophic costs has been estimated at 40%.^5^ The COVID-19 pandemic and associated economic crisis (alongside the ongoing conflict), could have further exacerbated the TB catastrophic cost. Nevertheless, nationally representative TB patient cost surveys have not been conducted. This study aimed thus to determine the magnitude and main drivers of costs incurred by TB patients and their households.

We conducted a health facility-based cross-sectional survey from November 2020 to February 2021 in 19 study zones in four regions in Ethiopia: Oromia, Amhara, Sidama, and Southern Nations, Nationalities and Peoples (SNNP). An estimated 80,481,771 people reside in these regions, representing 80% of the national population in 2020^6^ and contributing to 54% of the national TB burden.^7^ One-third of the public health facilities that provide TB treatment were randomly selected from all the districts within the study zones. The participants were selected randomly among patients receiving treatment during the study period, one from low TB load health facilities (<100 TB patients per year) and two from high TB load health facilities (<100 TB patients per year). TB patients who were receiving TB treatment in the sampled health facilities for at least 14 days, regardless of age and type of TB, were considered for inclusion in the study. The WHO generic TB cost survey questionnaire^8^ was customized to the local context and translated into three local languages (Amharic, Oromiffa and Sidama). Data were collected from patients or their parents/guardians while attending their TB treatment follow-up visits. The analysis included basic descriptive statistics to present sociodemographic factors and the extent of catastrophic costs. The average spending during the continuation phase was used to estimate the cost for the whole TB treatment period and of patients in the initiation phase. Catastrophic costs were determined by giving a binary value for whether patients incurred catastrophic costs or not. Bivariate and multivariate analyses were applied to assess the factors associated with TB patients’ catastrophic costs. All variables with a *P*-value of less than 0.05 in the bivariate analysis were entered into the multivariable regression. We obtained support letters and permission from each study region’s ethical review committee. Oral consent was obtained prior to the interview of TB patients. Assent was obtained from the parent or guardian for children under 14 years of age.

A total of 433 TB patients participated: 56% were male, the mean age was 33.6 (male: 35.8 and female: 30.8), the median age was 30 (male 33 and female 28), 59.4% had attended school grades 1–12, and 39.3% were farmers. One average, a TB patient incurred a total of USD715.5 for TB-related costs: with 87.8% and 12.2% direct and indirect costs (income lost related to time lost), respectively. Direct costs attributed to non-medical (food, drink, and accommodations) (55.5%), transport (20.7%) and medical cost for TB treatment (11.6%). Of the 433 study participants, 66.1% experienced catastrophic costs related to TB diagnosis and treatment when using a threshold of more than 20% of their annual income. Additionally, 39% faced catastrophic costs at a higher threshold of more than 30% of their annual income. Compared with TB patients from Amhara, Sidama TB patients were 71% less likely to face TB-related catastrophic costs (adjusted odds ratio [aOR] 0.29, 95% confidence interval [CI] 0.11–0.76). Farmers were 74% (aOR 0.26, 95% CI 0.11–0.62) more likely than government employees and 66% (aOR 0.34, 95% CI 0.18–0.61) more likely than the self-employed to experience catastrophic costs. TB patients with directly observed treatment (DOT) at home were 61% less likely to experience catastrophic costs than those who attended DOT at a health facility (aOR 0.39, 95% CI 0.24–0.65). TB patients with at least one family member on TB treatment were 93% more likely to experience catastrophic costs than those with no family member on TB treatment (aOR 1.93, 95% CI 1.10–3.42; Table).

**Table.** TB-related costs, catastrophic costs (>20% of household annual income), and bivariate and multivariable analysis based on the sociodemographic characteristics of TB patients (*n* = 433).

**A)** Sociodemographic characteristics of TB patients				
Variables	Not catastrophic	Catastrophic	Bivariate analysis	Multivariable analysis
	*n* (%)	*n* (%)	cOR (95% CI)	aOR (95% CI)
Region				
Amhara	43 (40)	64 (60)	1	
Oromia	76 (47.8)	83 (52.2)	0.73 (0.45–1.21)	0.65 (0.39–1.09)
Sidama	15 (65.2)	9 (34.8)	0.36 (0.14–0.92)	0.29 (0.11–0.76*)
SNNP	79 (55.2)	64 (44.8)	0.54 (0.33–0.90)	0.61 (0.36–1.04)
Sex				
Female	66 (34.4)	126 (65.6)	1	
Male	81 (33.6)	160 (66.4)	1.03 (0.70–1.54)	
Age, years				
<15	6 (40)	9 (60)	1	
15–24	35 (23.5)	76 (68.5)	1.45 (0.48–4.38)	
25–49	78 (32.6)	161 (67.4)	1.38 (0.47–4.00)	
>49	28 (41.2)	40 (58.8)	0.95 (0.30–2.98)	
Education				
Illiterate	49 (35.5)	89 (64.5)	0.86 (0.57–1.35)	
≤Grade 12	84 (32.6)	174 (67.4)	1	
>Grade 12	14 (37.8)	23 (62.2)	0.77 (0.38–1.61)	
Occupation				
Farmer	48 (28.6)	122 (71.8)	1	
Government employment	20 (45.5)	24 (54.6)	0.47 (0.24–0.93)	0.29 (0.1–0.66*)
Self-employed	50 (44.3)	63 (55.8)	0.5 (0.30–0.82)	0.44 (0.20–0.68*)
Unemployed	29 (27.4)	77 (72.6)	1.04 (0.61–1.80)	0.73 (0.39–1.40)
Treating health facility				
Private facility	53 (47.3)	59 (52.7)	1	
Public health center	31 (52.5)	28 (47.5)	0.52 (0.14–1.92)	
DOT support provider				
Health facility	77 (30)	170 (70)	1	
Self or home-based	70 (39.6)	107 (60.4)	0.44 (0.28–0.69)	0.39 (0.24–065*)

**B)** TB-related direct and indirect costs					
	Direct costs (USD)			Indirect costs (USD)	Total costs (USD)
	Transport costs	Drink, food and accommodation	Medical cost of TB treatment	Income lost related to time lost	
Cost in number	148.1	397.1	83.0	87.3	715.5
Proportion of total cost, %	20.7	55.5	11.6	12.2	100

* Statistically significant.

cOR = crude odds ratio; CI = confidence interval; aOR = adjusted OR; SNNP = Southern Nations, Nationalities and Peoples; DOT = directly observed therapy; USD = US dollar.

Our findings indicated approximately two-thirds of TB patients and their families experienced TB-related catastrophic costs. This is higher than the 2014 estimate of 58% across all low- and middle-income countries,^9^ 43% of global estimates,^10^ and 40% from Ethiopia^5^ and other African^9^ and Asian countries.^3^ This is despite the exempted cost of TB services in Ethiopia, with direct costs contributing the highest share. The prohibitively high direct non-medical costs, and medical costs representing less than 20%, have been reported by many studies conducted in Ethiopia^11^ and other African countries^12^ and systematic reviews.^9^ Therefore, the policy in Ethiopia may consider implementing a 'TB patients’ exemption' rather than a 'TB service exemption'. Receiving TB DOT at home is associated with a lower likelihood of experiencing catastrophic costs, which may be due to the reduced costs for transportation, food and accommodation associated with at-home treatment with fewer visits to health facilities.^13^ Further decentralization of DOT services near the patient, through self or home-based DOT, may reduce catastrophic costs. The higher financial impact of TB on farmers has repercussions often extending beyond the patient to the family and caregivers. Farmers often reside in rural areas, requiring them to overcome many barriers to adhere to DOT, such as travelling long distances to facilities.^2^ The regional differences in catastrophic costs are mainly due to differences in the type of TB: the Sidama region has a higher number of bacteriologically confirmed TB cases, whereas Ahmara has higher extrapulmonary TB.^14^ The diagnostic and treatment service for extrapulmonary TB is usually delayed, expensive and requires a longer period of treatment.^15^

In conclusion, the high burden of catastrophic costs necessitates a decentralization of TB diagnosis and treatment services, the scaling up of community and home-based DOT, and the need to establish a community support system to help with nutrition and transportation costs.
